# *In vivo* evaluation of the protective effects of arjunolic acid against lipopolysaccharide-induced septic myocardial injury

**DOI:** 10.7717/peerj.12986

**Published:** 2022-02-16

**Authors:** Hany Elsawy, Mohammed Almalki, Omar Elmenshawy, Ashraf Abdel-Moneim

**Affiliations:** 1 Department of Chemistry, Faculty of Science, King Faisal University, Al-Ahsa, Saudi Arabia; 2 Department of Chemistry, Faculty of Science, Tanta University, Tanta, Egypt; 3 Department of Biological Sciences, Faculty of Science, King Faisal University, Al-Ahsa, Saudi Arabia; 4 Department of Zoology, Faculty of Science, Al Azhar University, Cairo, Egypt; 5 Department of Zoology, Faculty of Science, Alexandria University, Alexandria, Egypt

**Keywords:** Endotoxin, Cardiotoxicity, Arjunolic acid, Oxidative stress, Cytokines, Caspase-3/8/9

## Abstract

Lipopolysaccharide (LPS) is a glycolipid component of the cell wall of Gram-negative bacteria, which induces multiple organ dysfunctions, eventually leading to septic shock and death. Arjunolic acid (AA) has been shown to have therapeutic benefits against various organ pathophysiologies, although its role in sepsis remains unclear. Here, we evaluated the effects of AA on LPS-induced free radical production and cardiotoxicity. Male albino mice were allocated to four groups: normal, 1.5 µg/30 g b.w. of LPS (LPS), 20 mg/kg b.w. AA with LPS (AA+LPS) and 20 mg/kg b.w. of AA (AA). Subsequently, blood and heart samples were harvested for biochemical and histopathological examinations. Pretreatment with AA attenuated LPS-induced increased serum levels of cardiac troponin I, lactate dehydrogenase and creatine kinase. In the meantime, AA pretreatment before LPS resulted in a significant increase in endogenous antioxidants (superoxide dismutase, catalase, glutathione peroxidase and reduced glutathione) and a significant decrease in the level of lipid peroxidation product (malondialdehyde) in the heart as compared to the LPS group, while cardiac cytochrome c activity were significantly increased. In addition, in the AA-pretreated mice, C-reactive protein and proinflammatory cytokines (interlukin-1 and tumor necrosis factor-alpha) were significantly reduced, and anti-inflammatory cytokines (interleukin-4 and -10) were significantly increased in cardiac tissues as compared to the LPS-treated animals. Furthermore, prior administration of AA to LPS exposed mice led to a significant a significant decrease in heart caspase-3, -8, and -9 as compared to the LPS group. Interestingly, AA was also able to improve LPS-induced histopathological changes in the cardiomyocytes. In conclusion, these *in vivo* findings indicate that AA may be a promising cardioprotective agent against LPS-stimulated cardiotoxicity, at least in part, through upregulation of cardiac antioxidants, reduction of lipid peroxidation, and inhibition of inflammation and cardiac cell death.

## Introduction

Sepsis is the leading cause of death in critical care medicine with an incidence steadily increases every year (Schulte, Bernhagen & Bucala, 2013). Sepsis-related organ failure is extremely complex, involving dysregulated systemic inflammation (Spoto, Valeriani & Costantino, 2015), caused by invading microorganisms or their products (Shimaoka & Park, 2008). Lipopolysaccharide (LPS), also known as lipoglycans or endotoxin, is the dominant cell wall molecule of the Gram-negative bacteria, contributing to septic inflammatory reaction (Kheiry et al., 2019), which in turn could lead to an oxidative stress status (Sebai et al., 2009). Increasing evidence demonstrates that LPS is one of the main triggers for the proinflammatory and pathophysiological responses seen in patients with septic shock (Zweigner et al., 2001; Wang et al., 2019). Heart dysfunction is a consequence of sepsis, and recent studies have demonstrated that LPS can induce inflammation and apoptosis in cardiomyocytes (Yang et al., 2016; Di et al., 2020). Indeed, sepsis-induced myocardial injury is usually correlated with features of decreased cardiac contractility, diastolic impairment, and cardiac injury, leading to a hypotensive symptoms (Drosatos et al., 2015; Kakihana et al., 2016). Polymicrobial sepsis may initiate myocardial cell depression *via* release of potent proinflammatory cytokines such as tumor necrosis factor (TNF)-α, interleukin (IL)-1β, and IL-6, which act as cardiodepressant mediators resulting in cardiac contractile dysfunction (Yousif et al., 2018).

Plant-derived natural products may help to protect against numerous cardiovascular diseases and disorders (Shah et al., 2019). Among these, arjunolic acid (AA), a naturally occurring chiral triterpenoid saponin, has been found in *Terminalia arjuna*. Previous studies have demonstrated that AA has powerful anti-inflammatory (Alyoussef, 2015) and antioxidant (Manna, Sinha & Sil, 2007; Pal et al., 2015) effects. In addition, AA has been shown to possess some cardioprotective properties (Sumitra et al., 2001; Manna, Sinha & Sil, 2008; Al-Gayyar et al., 2014). Despite its potential benefits on the heart, the cardioprotective effects of AA following exposure to septic challenge has never been investigated. To date, no therapy of septic cardiomyopathy, a key contributor to organ dysfunction in sepsis, is available globally for clinical use. Therefore, the present study aims to explore that hypothesis that AA may protect against LPS-induced cardiotoxicity in mice with sepsis. More specifically, we investigated the effect of AA on antioxidant capacity, inflammatory factors, and apoptotic caspases, along with myocardial pathological features.

## Materials and Methods

### Chemicals

AA (Cat. No. CFN 98690) was purchased from AOBIOUS, Inc., MA, USA. LPS (Cat. No. l3023) was obtained from Sigma-Aldrich, Germany. Other chemicals used were of analytical grade.

### Animals

The laboratory work was conducted at King Faisal University (Al-Ahsa, Saudi Arabia). The animal management and handling procedures were approved and licensed by the research ethics committee at King Faisal University (reference number: KFU-REC/2021-04-46 dated 29.4.2021). Male albino mice were obtained from an animal facility at the Faculty of Science, King Saud University, Saudi Arabia. Mice (20–25 g) were kept in stainless steel wire-bottomed cages, placed in a well-ventilated animal house and maintained at room temperature of 25 ± 2 °C with a natural 12-h light:dark cycle. All mice had fed access to chow and water during the study period. Extensive efforts have been done to minimize suffering of the animals in this research.

### Experimental design

After 2 weeks of acclimatization, the animals were randomly assigned into four groups, each consisted of five mice and they were treated according to the following schedule:

– Group I (normal control): animals received an intraperitoneal (i.p.) injection of vehicle (1% DMSO/saline) for 4 days.
– Group II (LPS): animals received an i.p. single dose of LPS (1.5 µg/30 g b.w).
– Group III (AA plus LPS): animals received an i.p. dose of AA (20 mg/kg b.w.) for 4 days plus a single i.p. dose of LPS (1.5 µg/30 g b.w) at the 4th day.
– Group IV (AA): animals received an i.p. dose of AA (20 mg/kg b.w.) for 4 days.

The dose of AA (Sinha, Manna & Sil, 2008) and LPS (Durgha et al., 2016) were selected based on previous studies. After 5–6 h of the last injection, the animals were sacrificed by cervical dislocation, blood was collected from trunk vessels, and then the serum was separated and stored at −20 °C until further use. The hearts were excised and the left ventricle tissue samples were obtained. The upper part of left ventricle was fixed in 10% neutral-buffered formalin solution for histopathological processing. The remaining heart tissues were immediately frozen at −80 °C for subsequent biochemical tests. Cardiac tissues were homogenized using glass homogenizer in 100 mM phosphate buffer containing 1 mM EDTA, pH 7.4 and centrifuged at 6000 rpm for 30 min at 4 °C. The supernatant was gathered and used for assay determinations.

### Determination of serum markers of heart injury

The level of cardiac troponin I (cTnI) was quantified using cTnI ELISA kit, (Cat. No. (SE120134) Sigma-Aldrich, MO, USA). The activities of lactate dehydrogenase (LDH) and creatine kinase (CK) were measured using colorimetric assay kits (LDH; Cat. No. ab 102526, CK; Cat No. ab 155901; Abcam Chemicals, Tokyo, Japan). LDH assay is based on the fact that LDH reduces NAD+ to NADH, which then interacts with a specific probe, producing a colour measured spectrophotometrically at 450 nm. The CK test principle depends on the conversion of creatine into phosphocreatine and ADP by the enzyme. The generated phosphocreatine and ADP react with CK enzyme mix to form an intermediate, which reduces a colorless probe to a coloured product with strong absorbance at 450 nm.

### Estimation of protein

The total protein concentrations of the heart homogenates were determined according to the method of Bradford (1976) using bovine serum albumin as a standard.

### Estimation of cardiac antioxidants and lipoperoxidation

Endogenous antioxidants like catalase (CAT), superoxide dismutase (SOD), glutathione peroxidases (GPx), and reduced glutathione (GSH) and lipid peroxidation product (malondialdehyde; MDA) were measured using commercial kits in accordance with the manufacturer instructions (CAT; Cat. No. CA 2517, SOD; Cat. No. SD 2521, GPx; Cat. No. GP 2529, GSH; Cat. No. GR 2511, MDA; Cat. No. MD 2529; Bio-Diagnostic, Giza, Egypt). CAT assay involves the measurement of the hydrogen peroxide (H_2_O_2_) substrate remaining after the action of CAT present in the sample. The rest of H_2_O_2_ was quantified after its conjugation with a chromogen. The intensity of the formed color was inversely proportional to the activity of CAT enzyme. SOD assay depends on its ability to inhibit the phenazine methosulphate-mediated reduction of nitroblue tetrazolium dye. The activity of GPx was determined as an indirect measurement, where the oxidized GSH formed by GPx was refreshed to its reduced form by glutathione reductase. Estimation of GSH is based on its reduction with 5,5′ dithiobis 2-nitrobezoic acid (DTNB). The content of GSH was directly proportional to the produced yellow color, which was measured at 405 nm. Lipid peroxidation (LPO) was assayed calorimetrically upon the reaction of MDA with thiobarbituric acid (TBA) in acidic medium at 95 °C. The produced pink product was detected at 534 nm.

### Cytochrome c oxidase assay in the heart tissues

Cytochrome c oxidase (CCO) is the terminal enzyme in the electron transport chain of the mitochondria and the main site of O_2_ consumption. The activity of CCO was quantified using CCO assay kit (Cat. No. CYTOCOX1; Sigma-Aldrich, MO, USA). The assay employs the decline in optical density of ferrocytochrome C measured at 550 nm, which is caused by its oxidation to ferricytochrome C by CCO.

### ELISA detection of heart inflammatory factors

C-reactive protein (CRP) was quantified using CRP Rat ELISA kit (Cat. No. EK0978; Bosten Scientific, Marlborough, MA, USA) according to the manufacturer’s protocol. Tumor necrosis factor-alpha (TNF- α) was estimated using Human TNF alpha ELISA Kit (Cat. No. EA100365;l OriGene Technologies Inc., Rockville, MD, USA). Interleukin 1 (IL-1), interleukin 4 (IL-4) and interleukin 10 (IL-10) were determined using standard ELISA methods with commercialized kits (IL-1; Cat. No MBS012415, IL-4; Cat. No. MBS162452, IL-10; Cat. No. MBS764911; MyBioSource, Inc., San Diego, CA, USA).

### Estimation of apoptotic markers

The levels of caspase 3 (casp-3), caspase 8 (casp-8) and caspase 9 (casp-9) in the heart supernatants were determined according to the methods described in their relevant ELISA kits (casp-3; Cat. No. MBS763727; casp-8; Cat. No. MBS764040; casp-9, Cat. No. MBS765858; MybioSource, Inc., San Diego, CA, USA). Sandwish ELISA technology was the strategy used for the determination of all caspases in this study.

### Histopathological studies

The formalin-fixed heart tissues were dehydrated in ascending series of ethanol, and embedded in paraffin. Sections of 4 µm-thick were prepared using a rotary microtome, stained with H and E protocol (haematoxylin and eosin dye), and examined under light microscope (Nikon 80i, Japan). The severity of histological changes were graded as previously described (Amara et al., 2013): -, no damage: +, mild; ++, moderate; and +++, severe. Six slides were prepared from each mouse heart. All sections were assessed for the presence of edema, inflammatory features, and myocardial cell apoptosis in a blinded fashion.

### Statistical analysis

Data was analyzed using SPSS version 16.0 software (SPSS Inc, Chicago, ILL Company). Statistical differences among groups were determined using one-way ANOVA followed by post hoc (Tukey) test. A *p* < 0.05 was considered to indicate a statistical significance.

## Results

### Cardiac marker levels

**Figure 1:**
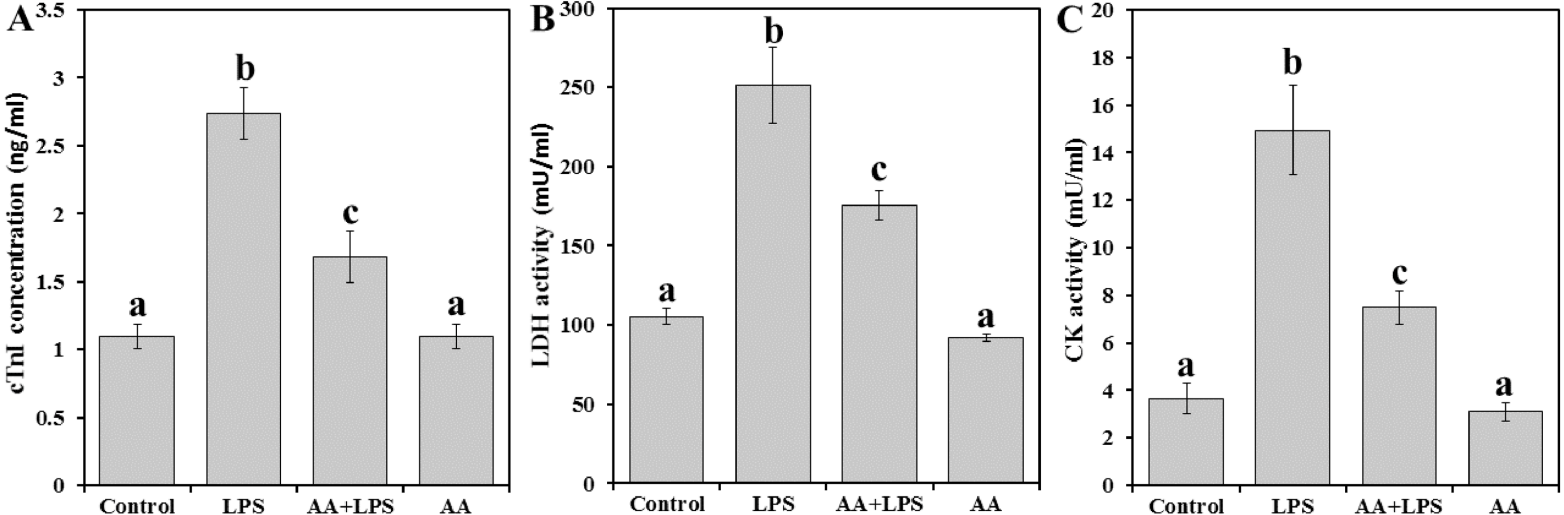
Markers of heart cell damage in mice serum from all experimental groups. Each bar represents mean ± SE, *n* = 5/group. Mice were pretreated with AA (20 mg/kg b.w.) for 4 days, and then challenged with a single dose of LPS (1.5 µg/30 g b.w). One way ANOVA is followed by LSD as a post hoc test. Mean values with different superscript letters are significantly different, *p* < 0.05, whereas means with the same superscript letter are not significantly different, *p* > 0.05.

We examined the activities of serum cardiac diagnostic marker enzymes like cTnI (Fig. 1A), LDH (Fig. 1A) and CK (Fig. 1C) in experimental mice. Levels of cTnI, LDH, and CK were significantly increased by 2.5, 2.4, and 4.1 folds, respectively in LPS group compared to control. Pretreatment with AA to LPS challenged mice significantly reduced serum cTnI (−38.7%), LDH (−30.1%), and CK (−49.9%) compared to LPS group, but still also significantly higher than the control levels.

### Heart-tissue redox status and CCO activity

**Figure 2:**
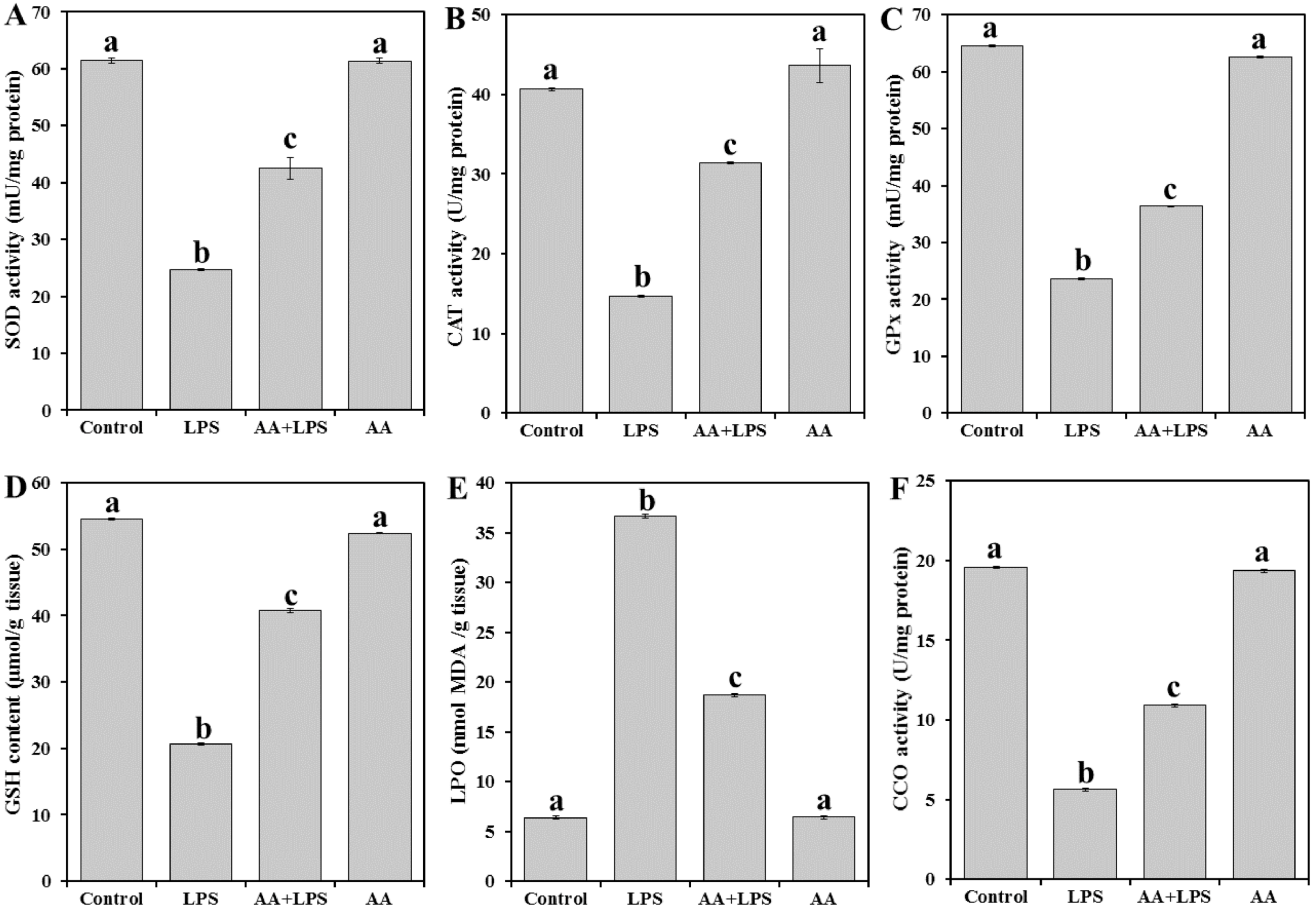
Levels of antioxidant parameters, MDA and CCO in mice heart from all experimental groups. Each bar represents mean ± SE, *n* = 5/group. Mice were pretreated with AA (20 mg/kg b.w.) for 4 days, and then challenged with a single dose of LPS (1.5 µg/30 g b.w). One way ANOVA is followed by LSD as a post hoc test. Mean values with different superscript letters are significantly different, *p* < 0.05, whereas means with the same superscript letter are not significantly different, *p* > 0.05.

To evaluate the oxidative stress parameters in the exposed animals, we measured enzymatic antioxidant SOD (Fig. 2A), CAT (Fig. 2B), and GPx (Fig. 2C), the content of non-enzymatic GSH (Fig. 2D) and the by-product of LPO (MDA) (Fig. 2E) in the heart tissue. LPS significantly decreased the levels of SOD (− 59.8%), CAT (− 63.8%), GPx (− 63.4%), and GSH (− 62.2%) and increased MDA production (+ 473.7%) when compared with the normal control. Pretreatment of AA to LPS-treated animals significantly elevated the levels of SOD (+ 72.1%), CAT (+ 94.4%), GPx (+ 54.1%) and GSH (+ 97.3%) and lowered the level of MDA (− 49.0%) when compared with the LPS-alone treated group, although the effect did not attain the control-like values.

As shown in Fig. 2F, the activity of CCO was significantly reduced in LPS group (− 71.2%) when compared with the control group of mice. AA treatment increased the level of CCO (+93.6%) as compared to LPS alone treated group but did not reach the control level.

### Heart inflammatory response

**Figure 3:**
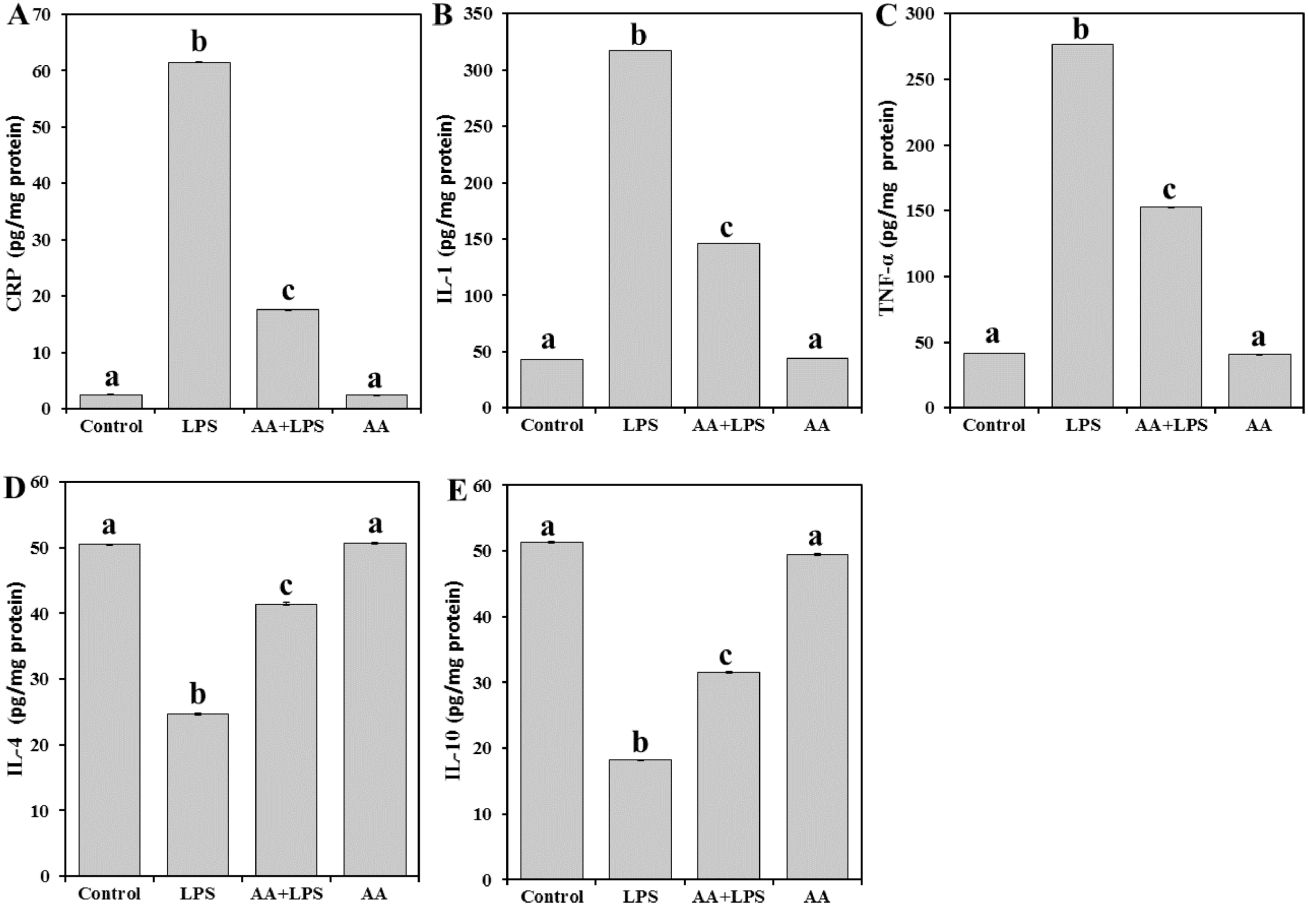
Levels of CRP, IL-1, TNF-α, IL-4 and IL-10 in mice heart from all experimental groups. Each bar represents mean ± SE, *n* = 5/group. Mice were pretreated with AA (20 mg/kg b.w.) for 4 days, and then challenged with a single dose of LPS (1.5 µg/30 g b.w). One way ANOVA is followed by LSD as a post hoc test. Mean values with different superscript letters are significantly different, *p* < 0.05, whereas means with the same superscript letter are not significantly different, *p* > 0.05.

Next, in order to explore the effect of AA on the tissue inflammatory mediators, the levels of CRP (Fig. 3A), and cytokines such as IL-1 (Fig. 3B), TNF- α (Fig. 3C), IL-4 (Fig. 3D), and IL-10 (Fig. 3E) were determined. There was a significant increase in the levels of CRP (by 24.6-fold) and proinflammatory IL-1 (by 7.3-fold), and TNF- α (by 6.7-fold) in the LPS group. In contrast, a significant decline in the levels of antiinflammatory IL-4 (− 51.1%), and IL-10 (− 64.6%) was observed. Pretreatment with AA significantly decreased the elevated levels of CRP (− 71.6%), IL-1 (− 53.9%), and TNF- α (− 44.8%) and restored the depleted IL-4 (+ 68.1%) and IL-10 (+ 73.3%) in the LPS-intoxicated mice. Yet, AA+LPS group did not reach the control values.

### Cardiac cell apoptosis

**Figure 4:**
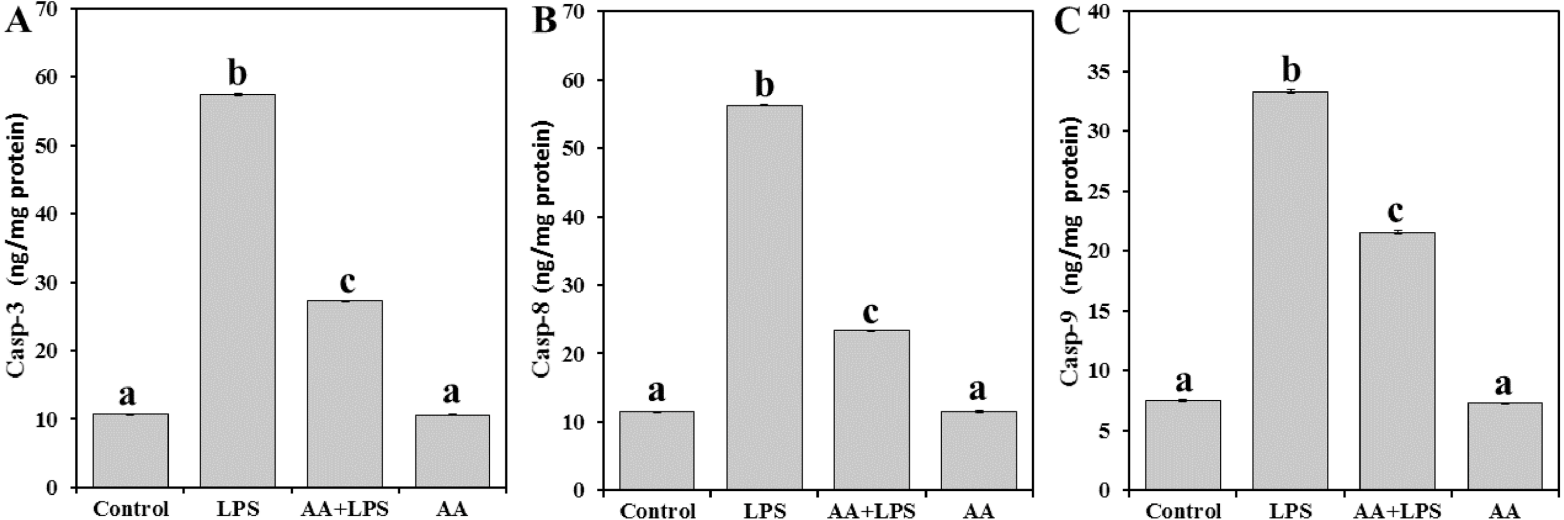
Levels of caspase-3, -8 and -9 in mice heart from all experimental groups. Each bar represents mean ± SE, *n* = 5/group. Mice were pretreated with AA (20 mg/kg b.w.) for 4 days, and then challenged with a single dose of LPS (1.5 µg/30 g b.w). One way ANOVA is followed by LSD as a post hoc test. Mean values with different superscript letters are significantly different, *p* < 0.05, whereas means with the same superscript letter are not significantly different, *p* > 0.05.

In order to address the mechanism underlying the AA-mediated protection of mouse myocardial tissue from LPS, casp-3 (Fig. 4A), casp-8 (Fig. 4B), and casp-9 (Fig. 4C) were examined. LPS was demonstrated to increase the levels of casp-3 (by 5.4-fold), casp-8 (by 4.9-fold) and casp-9 (by 4.5-fold), as compared with the control mice. However, pretreatment of AA reduced the levels of casp-3 (− 52.5%), casp-8 (− 58.5%) and casp-9 (− 35.3%) when compared with the LPS-alone group, without returning to control group levels.

### Cardiomyopathy

**Figure 5:**
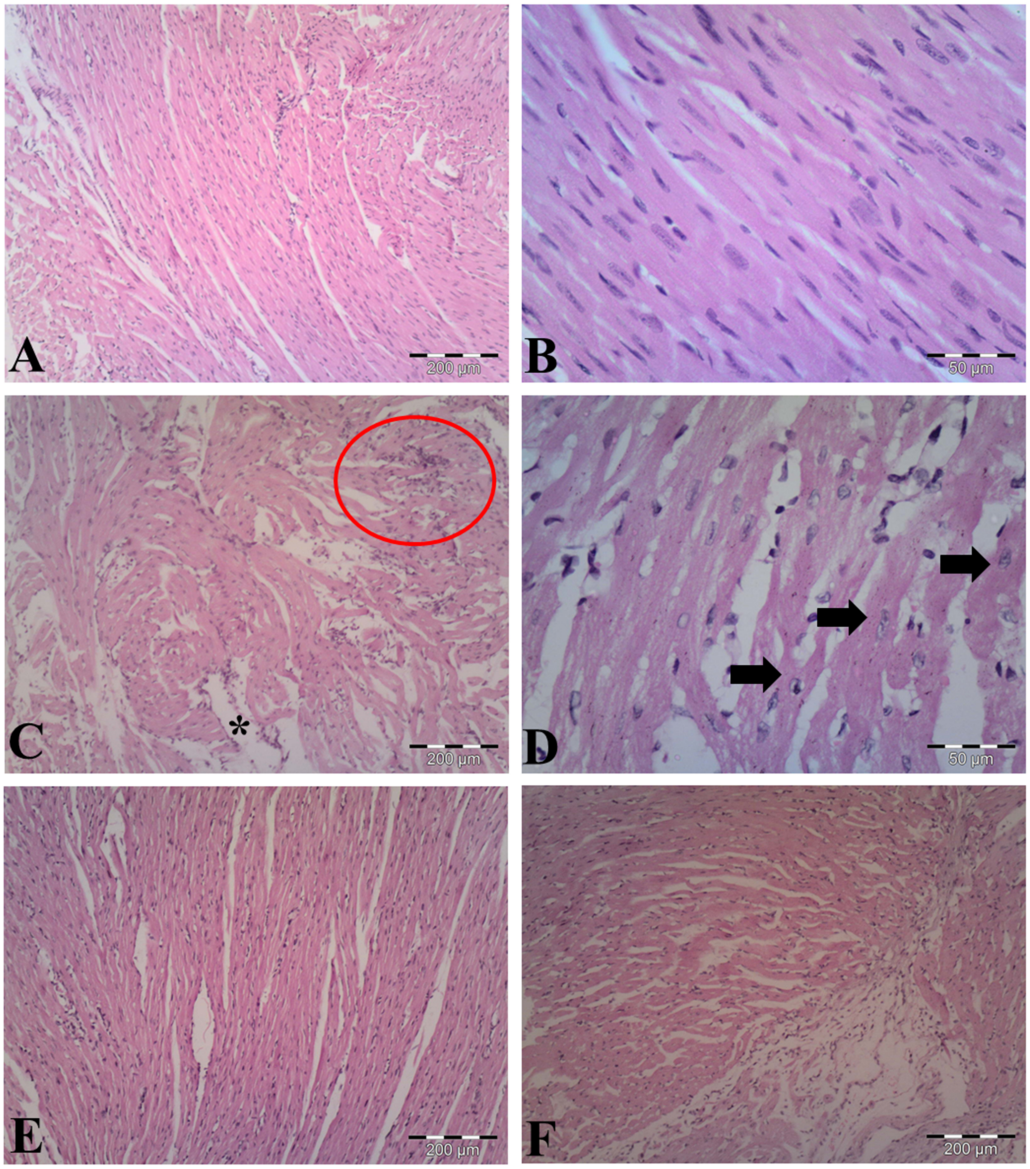
Histological findings in heart tissue of adult mice from all experimental groups. Mice were pretreated with AA (20 mg/kg b. w.) for 4 days, and then challenged with a single dose of LPS (1.5 µg/30 g b.w). (A, B) Vehicle-control group shows normal myocardial fibers with no signs of apoptotic cell death. (C, D) LPS group displays disrupted architecture, edematous intramuscular space (*) and marked infiltration of inflammatory cells (red circle) (in C), and apoptotic cells (indicated by arrows) (in D). (E) AA+LPS group demonstrates the decrease in damages. (F) AA group depicts normal muscle fibers without any pathological changes. Hematoxylin-eosin stain, scale bar: 200 µm (A, C, E, F), 50 µm (B, D).

The observations of myocardial histoarchitecture are graded and summarized in Table 1. H&E-stained heart sections from vehicle-control group indicated normal cardiomyocyte structures (Figs. 5A, 5B). As shown in Figs. 5C & 5D, LPS induced edematous intramuscular space, focal inflammatory infiltrates and apoptotic cells, showing atrophied nucleus and cytoplasmic eosinophilia, in the heart of LPS-treated mice. Pretreatment with AA in LPS group reduced myocardial damages (Fig. 5E), suggesting that AA exerted protective effect in this model of cardiac injury. The AA only treated mice exerted no significant changes in the myocardial structure (Fig. 5F).

**Table 1:** Grading of the histopathological changes in the myocardium in different experimental groups.

Treatments	Edema	Inflammatory infiltrations	Apoptosis
Control	−	−	−
LPS	+++	+++	+++
AA+LPS	+	++	++
AA	−	−	−

**Notes.**

Scoring was done as follows: none (−), mild (+), moderate (++) and severe (+++) damage.

## Discussion

The current study investigated the protective role of AA against the cardiotoxic effects of LPS in mice. The major findings of the current study demonstrated that pretreatment with AA could effectively counteract septic cardiomyopathy *via* suppression of oxidative stress, myocardial inflammatory injury and caspase-dependent cell death. Our results have shown that LPS injection induced deleterious heart lesions as pointed out by elevated levels of cTnI, LDH and CK. These observations are in line with several studies, which substantiated the marked cardiac membrane damage associated with LPS exposure (Ammann et al., 2001; He et al., 2015; Zhang et al., 2021). Thus, a reduction in cardiac markers by AA reflects its ability to maintain the integrity of cell membrane (Hemalatha et al., 2010).

Oxidative stress is thought to be one of the major causatives for various diseases and drug-induced organ toxicity (Pereira et al., 2012). In our study, endotoxin/LPS induced oxidative stress markers in an in *vivo* model as demonstrated by the decreased cardiac antioxidant capacities and the increased MDA level. Our results were in agreement with other reports (Goraca, Piechota & Huk-Kolega, 2009; Plotnikov et al., 2017; Ahmadabady et al., 2021). In fact, the local increase of MDA indicates reactive oxygen species (ROS)-dependent tissue damage (Kwiecien et al., 2014). There is evidence that during sepsis, myocardial dysfunction may result from increased production of ROS from cardiomyocyte mitochondria (Tsolaki et al., 2017). ROS byproducts generated from the mitochondria further stimulates ROS production in endothelial cells, resulting in an amplified free radical signal and pathological oxidative stress damage (Murphy, 2009). Moreover, using a septic mice model, it was shown that ROS derived from NOX1/NADPH oxidase induced cardiomyocyte apoptosis by increasing oxidation of Akt and subsequent dephosphorylation by protein phosphatase 2A, while NOX1 deficient animals displayed reduced rates of apoptosis (Matsuno et al., 2012). Many studies have shown that AA could prevent LPO (Manna, Sinha & Sil, 2007; Pal et al., 2015), a crucial factor contributing to the progression of many diseases (Negre-Salvayre et al., 2008). AA has one primary and two secondary hydroxyl groups, therefore, it can be expected that like vitamin C, AA could also remove ROS and other intracellular oxidants from the medium that are largely responsible of cell LPO (Hemalatha et al., 2010). Al-Gayyar et al. (2014) assumed that AA protects against sodium nitrite-produced cardiotoxicity in Sprague-Dawley rats, through a reduction in parameters indicating LPO. Although AA possesses free radical scavenging activities (Manna, Sinha & Sil, 2008), we observed partial reversal (but not to the normal levels) of cardiac MDA and the antioxidant indices that were altered in LPS-induced oxidative stress in septic heart. Enzymatic and non-enzymatic antioxidants at the appropriate cellular locale shield against ROS in conditions with increased oxidative stress (van der Pol et al., 2019). It is apparent that protection against LPS may have been mediated by an elevation in myocardial antioxidant defenses induced by AA.

On the other hand, there are reports suggesting that increased ROS overproduction under oxidative stress may reduce mitochondrial respiratory enzyme activities (*e.g.*, CCO) with further disruption of ATP production and enhanced cell apoptosis (Zapelini et al., 2008; Quoilin et al., 2014). However, our results indicated that AA treatment appears to protect against the oxidative modification of cardiac CCO and helped in preserving the mitochondrial function. Al-Gayyar et al. (2014) reported similar finding in another model of cardiovascular burden.

Then, we studied cytokine response levels in response to LPS exposure. There is a growing appreciation for the importance of inflammation, in addition to direct cytotoxicity, in the cardiomyopathy during sepsis (Virzi et al., 2017). In the present study, endotoxin increased the level of pro-inflammatory cytokines (TNF-α and IL-1β) and reduced the level of anti-inflammatory cytokines (IL-4 and IL-10). Furthermore, the data obtained in our study revealed an increase in CRP level, the cardiac inflammatory marker after treatment with endotoxin. CRP has been shown to promote the production of pro-apoptotic cytokines and inflammatory mediators (such as IL-1 β, TNFα, and ROS) *via* the activation of Fc-γ receptors (Kobayashi et al., 2003; Devaraj, Du Clos & Jialal, 2005). AA has been shown to reduce the inflammatory response, thereby achieving marked cardioprotection in several *in vivo* models (Ghosh et al., 2010; Al-Gayyar et al., 2014; Sherif, 2015). Furthermore, AA has the ability to reduce vascular inflammation and prevent the activation of ROS-mediated signaling cascades that eventually lead to cell death (Manna et al., 2010). In the current study, pretreatment with AA not only significantly modulated the LPS-induced cardiac inflammatory dysregulation, but also lessened the extensive heart lesions caused by LPS exposure. These results suggest that AA provides a significant protection against endotoxin-induced heart morphological injuries.

Cardiomyocyte apoptosis are closely implicated in the pathogenesis of septic shock in animal models (Nezic et al., 2018). Activation of apoptotic caspases is centrally involved in endotoxin-induced cardiomyocyte contractile dysfunction, sarcomere disorganization, and perturbations of myofibrillar calcium pumps and channels (Schwab et al., 2002; Lancel et al., 2005). During sepsis, casp-8 can be activated by signaling complex *via* death receptors, like FAS ligand or TNF-α (Hotchkiss et al., 2005; Aziz et al., 2013). In turn, casp-8 cleaves cytoplasmic Bid into tBid, which then translocates to the mitochondria and activates a signaling cascade of downstream proteases including casp-3, and -9, leading to apoptosis (Chung et al., 2010). In our experiment, AA was found to alleviate elevated activities of casp-3, -8 and -9 in the heart tissues of LPS-treated mice. AA has previously found to effectively counteract doxorubicin-induced cardiac apoptosis *via* inhibiting ROS-dependent activation of JNK-p38 and p53-mediated signalling pathways (Ghosh et al., 2011). AA supplementation exerts an anti-apoptotic action in hyperglycemia-induced cardiovascular complication through downregulation of caspase-dependent pathway (Ghosh & Sil, 2013). In addition, AA attenuated both mitochondrion-dependent and independent apoptotic cell death in acetaminophen-induced nephrotoxicity (Ghosh et al., 2010) and cadmium-induced liver damage (Pal et al., 2011). So, we can suggest that AA may be useful in ameliorating endotoxin cardiotoxicity through its anti-apoptotic effect.

The present study has some limitations that need to be addressed later. First, it may be beneficial to include electrocardiogram (ECG) measurements. Second, AA-related mechanism and therapy should be worthy explored at the molecular level. However, it is important to highlight that this is the first study to analyze the effects of AA on cardiac oxidative stress in septic model mice. Our results, therefore, are valid only within the scope of the proposed model, and at this preliminary stage, the provided data are not expected to supply any clinically useful information.

In conclusion, AA results in partial protection against LPS induced septic cardiotoxicity by (1) improving myocardial redox state and endogenous antioxidant reserve; (2) blocking of LPS-induced heart inflammatory injury; (3) reducing LPS-induced activation of proapoptotic casp-3/8/9. To further determine the optimal doses of AA, future studies should test more doses/durations with emphasis on mitochondrial function and protein trafficking.

##  Supplemental Information

10.7717/peerj.12986/supp-1 Supplemental Information 1 Raw data

10.7717/peerj.12986/supp-2 Supplemental Information 2 ARRIVE 2.0 Checklist

## Additional Information and Declarations

### Data Availability

The following information was supplied regarding data availability: The raw data is available in the Supplemental File.
